# Empathy as a crucial skill in disrupting disparities in global brain health

**DOI:** 10.3389/fneur.2023.1189143

**Published:** 2023-12-15

**Authors:** Fasihah Irfani Fitri, Carmen Lage, Tatyana Mollayeva, Hernando Santamaria-Garcia, Melissa Chan, Marcia R. Cominetti, Tselmen Daria, Gillian Fallon, Dominic Gately, Muthoni Gichu, Sandra Giménez, Raquel Gutierrez Zuniga, Rafi Hadad, Tanisha Hill-Jarrett, Mick O’Kelly, Luis Martinez, Paul Modjaji, Ntkozo Ngcobo, Rafal Nowak, Chukwuanugo Ogbuagu, Moïse Roche, Cristiano Schaffer Aguzzoli, So Young Shin, Erin Smith, Selam Aberra Yoseph, Yared Zewde, Yavuz Ayhan

**Affiliations:** ^1^Department of Neurology, Faculty of Medicine, Universitas Sumatera Utara, Medan, Indonesia; ^2^Senior Atlantic Fellow at the Global Brain Health Institute/Trinity College, UCSF, Dublin, Ireland; ^3^Department of Neurology, Marques de Valdecilla University Hospital - Valdecilla Research Institute (IDIVAL), Santander, Spain; ^4^Senior Atlantic Fellow at the Global Brain Health Institute/Trinity College, UCSF, San Francisco, CA, United States; ^5^Canada Research Chairs, Ottawa, ON, Canada; ^6^The KITE Research Institute, Toronto Rehabilitation Institute, University Health Network, Toronto, ON, Canada; ^7^Dalla Lana School of Public Health, University of Toronto, Toronto, ON, Canada; ^8^Temerty Faculty of Medicine, University of Toronto, Toronto, ON, Canada; ^9^Acquired Brain Injury Research Lab, Department of Occupational Science and Occupational Therapy, Faculty of Medicine, University of Toronto, Toronto, ON, Canada; ^10^Center of Memory and Cognition Intellectus, Hospital Universitario San Ignacio Bogotá, Bogotá, Colombia; ^11^Pontificia Universidad Javeriana (PhD Program in Neuroscience) Bogotá, Bogotá, Colombia; ^12^Department of Social Sciences, University of Luxembourg, Luxembourg, Luxembourg; ^13^Department of Gerontology, Federal University of São Carlos, São Carlos, Brazil; ^14^Gladstone Institutes, San Francisco, CA, United States; ^15^Division of Geriatric Medicine at the Ministry of Health, Nairobi, Kenya; ^16^Multidisciplinary Sleep Unit, Memory Unit, Hospital de la Santa Creu i Sant Pau, Universitat Autònoma de Barcelona, Barcelona, Spain; ^17^Hospital Quirónsalud Valle del Henares, Madrid, Spain; ^18^Rambam Health Care Campus, Haifa, Israel; ^19^Memory and Aging Center, Medical Center, University of California, San Francisco, CA, United States; ^20^National College of Art and Design, Dublin, Ireland; ^21^Department of Psychiatry, University of KwaZulu-Natal, Durban, South Africa; ^22^Neuroelectrics (Spain), Barcelona, Spain; ^23^Faculty of Basic Clinical Sciences, Nnamdi Azikiwe University Teaching Hospital, Nnewi, Nigeria; ^24^Division of Psychiatry, UCL, London, United Kingdom; ^25^Department of Psychiatry, School of Medicine, University of Pittsburgh, Pittsburgh, PA, United States; ^26^College of Nursing, Inje University, Busan, Republic of Korea; ^27^Stanford University, Stanford, CA, United States; ^28^College of Health Sciences, Addis Ababa University, Addis Ababa, Ethiopia; ^29^Department of Psychiatry, Faculty of Medicine, Hacettepe University, Ankara, Türkiye

**Keywords:** empathy, brain health, cultural humility, disparity, equity, leadership, value

## Abstract

Brain health refers to the state of a person’s brain function across various domains, including cognitive, behavioral and motor functions. Healthy brains are associated with better individual health, increased creativity, and enhanced productivity. A person’s brain health is intricately connected to personal, social and environmental factors. Racial, ethnic, and social disparities affect brain health and on the global scale these disparities within and between regions present a hurdle to brain health. To overcome global disparities, greater collaboration between practitioners and healthcare providers and the people they serve is essential. This requires cultural humility driven by empathy. Empathy is a core prosocial value, a cognitive-emotional skill that helps us understand ourselves and others. This position paper aims to provide an overview of the vital roles of empathy, cooperation, and interdisciplinary partnerships. By consciously integrating this understanding in practice, leaders can better position themselves to address the diverse challenges faced by communities, promote inclusivity in policies and practices, and further more equitable solutions to the problem of global brain health.

## Introduction

In today’s interdependent world, prioritizing and improving brain health becomes a vital goal in advancing the collective health of populations across the globe (1, 2). The term “brain health” is not a standardized one. According to the World Health Organization (WHO), brain health is “the state of brain functioning across cognitive, sensory, social–emotional, behavioral and motor domains, allowing a person to realize their full potential over the life course, irrespective of the presence or absence of disorders” (3). There is heterogeneity among institutions and researchers on how brain health is defined, a recent literature review on definitions of brain health revealed that, despite these differences, the common thread is the conceptualization of brain health as dynamic and multidimensional, encompassing both objectively measured and perceived components (4).

**Figure 1:**
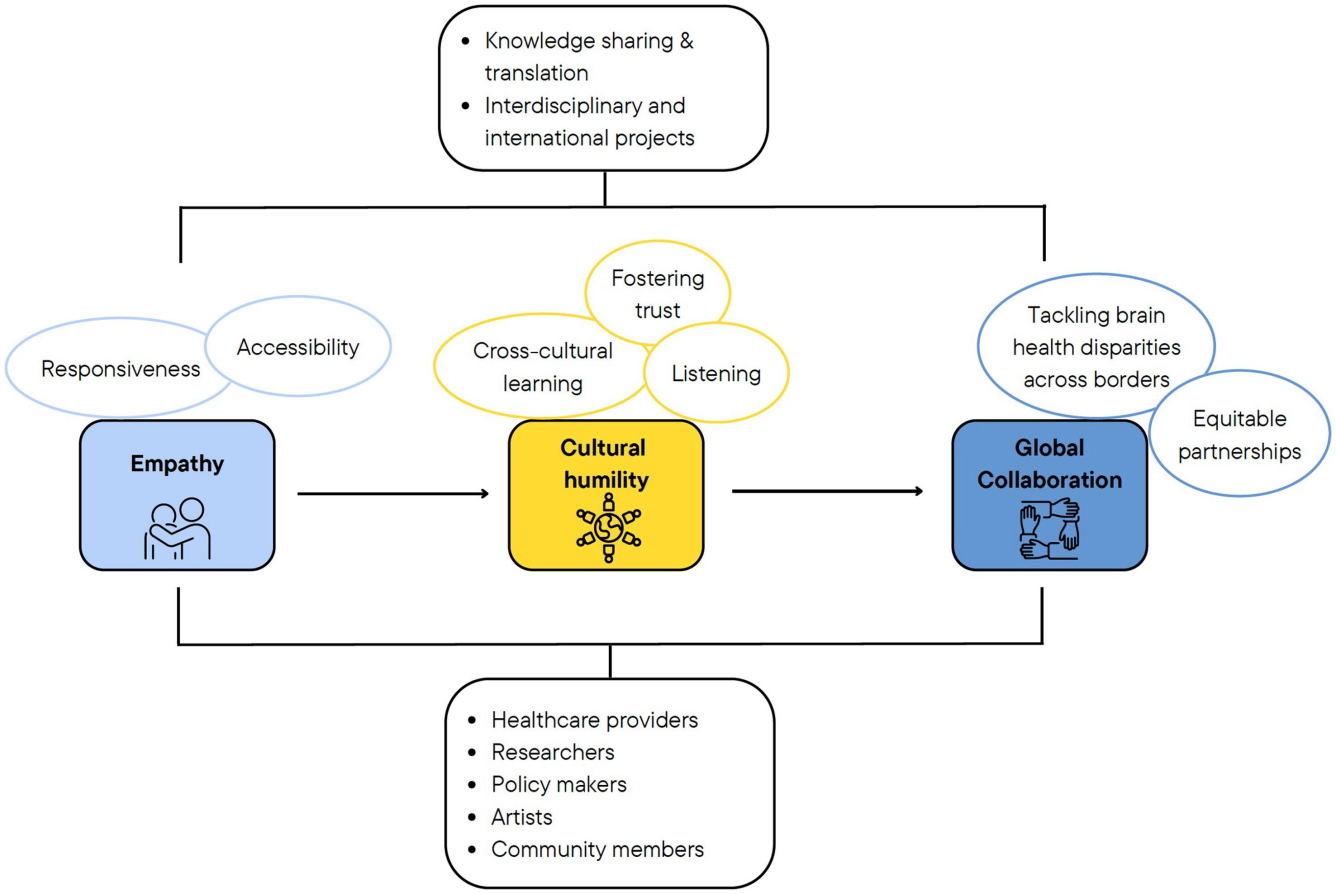
Framework outlining the position advanced in the paper. The value of empathy in tackling disparities in brain health by encouraging more responsive and accessible services, fostering trust among and between diverse populations through cultural humility, and pursuing collaborative interdisciplinary efforts to create responsive, accessible, and effective systems of care.

Progressively realizing the global right to brain health and global interdependence (i.e., worldwide mutual dependence between countries) allows systematically identifying and eliminating inequities stemming from social processes. Global leaders need to appreciate the effects of culture on dynamics between people and communities and to consciously commit to developing cultural humility. Cultural humility involves recognizing the values and perspectives of different cultures, advocating for inclusive and culturally sensitive approaches, and identifying the unique barriers that contribute to health and other social disparities. Empathy, a fundamental aspect of social interaction, a construct that accounts for a sense of similarity in feelings experienced by oneself and others, is essential to truly acknowledging the needs of others, for open conversation around challenging topics and for creative problem solving (Figure 1).

In this position paper, we argue that empathy, cultural humility and cooperation as fundamental for eliminating disparities and achieving global equity in brain health. We start by explaining inequalities and demonstrating disparities in global brain health. Then we identify cultural humility and emphasize its role in dealing with the global health problems. We focus on empathy and discuss how empathy and cooperation can challenge the biases and assumptions that underpin brain health disparities. Finally, we envision how art and science collaborations can remove barriers between individuals, institutions, and nations, and improve the prospects for equity in brain health across the world.

## Global inequalities affect the most vulnerable social groups

Health inequalities and health inequity have been defined in various ways with overlapping use (5). According to the World Health Organization (WHO), health inequalities are measurable differences in health across population subgroups, and health equity is defined as the absence of unfair, avoidable or remediable differences in health among population groups, defined by social, economic, demographic or geographic characteristics (6, 7). Different theories were developed to explain health inequalities. The inequalities might be associated with the differences based on culture, health behaviors, social mobility or genetics; however the main causal factor for the differences in health outcomes is suggested to be the differences in socioeconomic status of social groups, as proposed by the structural theory (8). This understanding calls for a more systematic way of approaching the concern of brain health inequalities for individuals and communities, as they perpetuate cycles of disadvantage and contribute to broader health inequities. The 2021 Global Health Security Index, an indicator of health security and related capabilities across 195 countries, analyzing experience from the COVID-19 pandemic, concluded that most countries came unprepared maintaining a capable accessible health system, where preparedness needs of vulnerable populations were most often neglected (9). Examples of vulnerable populations include people with cognitive and physical disabilities, chronic health conditions (e.g., diabetes, heart disease), and mental health issues (10). These vulnerabilities are also known risks for brain health across the world, including high, middle- and low-income countries.

## Understanding brain health disparities

Disparities in brain health are evident in different facets. Take the resource aspect as an example. The number of specialists and healthcare workers, the number of facilities per individual, and their resources respecting brain health widely vary across the globe. There is a clear lack of brain health workforce particularly in Africa and South East Asia (11, 12). This discrepancy remains when the workforce is classified by income level; low and lower-middle-income countries are in a very disadvantageous position. Disparities are overwhelming for brain health risk factors, which include but are not limited to education (13), smoking (14), diabetes (15), and hypertension (16). A brief survey on disparities in dementia among the authors of this paper from 17 countries and 5 continents displayed the existence of apparent inequalities in access to assessment and treatment [unpublished data, presented at the Global Brain Health Institute (GBHI) Annual Meeting 2022]. The surveyors found such disparities also existed within the countries and varied with social and economic factors.

Wealth, education, occupational status, race/ ethnicity or gender predict variations in health status on an individual level, these factors converge with a dynamic interdependent process influencing the health outcomes (17). Wealth disparities, measured according to the distribution of wealth from economic activity through jobs and demands, income, and pollution levels, exist both between and within countries (18). Disparities in the distribution of wealth across the globe are reflected in global peace (19). The 2021 Global Peace Index highlighted the connection between peace and health, emphasizing the role of disparities within and between nations associated with domestic and international conflicts, respectively (19). A responsible, ethical, and inclusive global health approach constitutes a genuine foundation to take action to promote brain health across the globe (20). The aim remains to disrupt disparities in brain health within and between countries. It is hard to imagine a more significant challenge for the leaders than to combat disparities.

## Disparities display the existence of global inequities in health

Disparities among different social groups remain a problem for brain health across the globe as exemplified in dementia. In the US, even though there is an overall decline in the disparities trend in dementia prevalence (21), racial and ethnic disparities remain significant (22–25). The annual incidence of dementia among African American and Hispanic American populations was significantly higher as compared to Caucasian population (26), and the burden of known risk factors for dementia differed among ethnic groups (27). The Global Council on Brain Health, a collaboration of experts convened by the AARP emphasizes the role of social determinants of health and identifies health disparities as a policy imperative (10, 28). The US National Plan to Address Alzheimer’s Disease (29) includes efforts to address inequities in dementia risk factors among vulnerable populations, based on needs assessment and developing targeted plans addressing any system gaps that stand in the way of such efforts. In Canada, the rates of dementia and its modifiable risk factors started to decrease, and protective factors increase, according to the 2021 annual report to Parliament on Canada’s national dementia strategy, A Dementia Strategy for Canada: Together We Aspire (30). The progress is attributed to collective aspirations embedded in the strategy’s national objectives including advancing knowledge of Canadians about modifiable risks of dementia, access to quality care, and raising awareness of, and elimination of stigmatizing behaviors. Despite these collective efforts, some populations within Canada have been identified as more likely to face barriers to equitable care and/or are at higher risk of developing dementia, including transgender and non-binary adults living with dementia, Indigenous people, and people from ethnic populations and those living in rural and remote communities (31, 32).

In the low – and middle income countries, socioeconomic factors including indices of poverty were associated with increased dementia rates in South Africa (33), Colombia (34), Chile (35), and Brazil (36). While dementia rates are exponentially growing in all countries of the world, and projections display that almost 150 million individuals will be affected by dementia by 2050 (37, 38), the estimations rates are disproportionately high for low to middle-income countries as compared to high income countries (39).

Unjustified and avoidable differences in race/ethnicity, gender, and class-based socioeconomic differences exist in many other neurological and mental health disorders across the globe (40–43), with disparities started to emerge during early stages of brain development (44). The effects of these individual factors on brain health may be distinct and particularly robust on individuals with intersectional identities. This is alarming and calls for action of global leaders for equity in brain health. It also recognizes the importance of investing in efforts to identify and discuss challenges related to disparities, identify opportunities for global collaboration and action, and share ideas for a global strategy.

## Overcoming disparities requires respectful dialog, and its essence is cultural humility

Effectively addressing global disparities in brain health requires collaboration among diverse groups of people from different cultural backgrounds. To ensure successful interactions, it is crucial to appreciate the cultural constructs and history of partners (45, 46).

Cultural humility refers to a more thoughtful and substantive understanding of other cultures and people which are unlike our own, and application of such understanding in practice. Tervalon and Murray-Garcia first coined the term “cultural humility” as a corrective to cultural competence, a skill that can be taught, acquired, and achieved, and it is frequently referred to as a necessary and sufficient condition for working effectively with diverse groups of people possessing ethnic, racial, and class differences (47). Cultural humility involves actively engaging in the process of learning about other cultures, their worldview, and any oppression or discrimination that they may have historically experienced, while also being mindful of our thoughts and feelings regarding those cultures. This includes overcoming any preconceived assumptions, prejudices, and biases people carry (45, 46).

Cultural humility calls for application in various aspects of life, including clinical work, education, and research. The need for cultural humility is particularly important in clinical practice, where clinicians are working with people from widely diverse cultural backgrounds. Putting aside their own belief systems and considering patients’ and caregivers’ perspectives is an essential skill for clinicians when developing a patient-centered treatment or care plan (48, 49). Inquiring about patients’ backgrounds, practices, religion, and culture, is essential to avoid stereotyping and in determining individual patient’s needs, goals, and preferable treatment options, to be in line with the paradigm of person-centered care. Cultural humility helps to create deeper connections and understanding between patients and care providers, which may increase patient satisfaction and care outcomes for vulnerable groups/minorities.

It is important to emphasize the role of cultural humility in identifying, addressing, preventing, and eliminating racism and discrimination in healthcare institutions (50–53). Racism in healthcare politics was suggested as a root cause of racial health inequities in the US (51). Discrimination may also be evident based on religion or other social factors. Historical attitudes toward the health care institutions, especially among marginalized communities, can shape their perceptions and health-related decisions (54, 55). Understanding and resolving underlying factors affecting trust in medical institutions play a crucial role in moderating the likelihood of individuals pursuing medical care (56, 57).

In research, especially in health research, the application of cultural humility allows the researchers to generate culture-sensitive hypotheses, apply culturally-neutral methods, and analyze the results with an appreciation of the influence of their own culture (58). Multicultural projects having a common goal but different resources in executing the same mutual interests have challenges. Each party has inherent differences, differing values, methods and rewards. Understanding the basic motivations, values, and sensitivities of each party in multicultural projects is crucial, as it lays the foundation for proper recognition and acknowledgment of their independence. This is essential because it fosters an environment of respect and inclusivity, allowing diverse groups to collaborate effectively toward a common goal. Acknowledging and respecting cultural differences becomes pivotal in international collaboration, as such recognition paves the way for meaningful communication and conflict resolution, promoting successful outcomes for all parties involved.

A troubling aspect in research is funding disparity for ethnic and racial minorities and women, and non-English speaking countries. Cultural humility allows us to reflect on the ways global funding and publishing agencies impact scientific research, and how scholars with cultures and languages other than English are frequently excluded due to biases or language barriers, and limited access to funding (58, 59).

Cultural humility is also essential in education, especially in disciplines emphasizing professional training and tied to a patient or client (e.g., nursing, medicine, and counseling). Humanities-based disciplines (e.g., art, music, literature, film, and theater) are well-positioned to incorporate cultural humility by addressing the perspectives of people in ways that encourage critical reflection, empathy, and appreciation for diversity in human artistic creativity (60).

### Art as a manifestation and media of cultural humility

Artistic manifestations are the fingerprints of a culture. Whether it is a painting, a dance, or a novel, artistic works are shaped by the character and the history of the culture in which they are created (61). Therefore, being exposed to artistic expressions can help us to understand a different culture and its particular perspective of the world (62). While this can help us recognize and embrace our own culture’s uniqueness, it can also help us appreciate others and embrace diversity. In the global health scenario, this means that art can be a tool to promote respectful intercultural dialog.

But, also importantly, art reflects our cultural past. In traditional artistic expressions, we can recognize the diverse influences that have carved a culture across time. We can also identify some of the same features in other - separated- cultures, realizing that they are not watertight compartments throughout history. This way, barriers between cultures begin to blur, our cultural arrogance smooths because we are aware of how much we share, and we start to recognize ourselves as habitants of one unique world, and not so different from our pairs.

Embracing cultural humility offers a valuable approach to navigating diversity and counter impediments to disparities that emerged from marginalization and stigmatization of disadvantaged communities. In light of disparities in brain health, cultural humility can foster empowerment, inclusivity, and respectful global collaborations.

## The role of empathy in brain health leadership

Leadership skills are required to promote public health changes to protect and promote brain health (63). Leaders must profoundly understand their own and global community (64–66), including cultural beliefs, practices, and political views, to create and implement reproducible, sustainable, and scalable interventions to protect brain health. Leaders need to create cross-culture connections to navigate global landscape, and to be in a position to influence people from diverse backgrounds. No problem will be solved without being connected with those who struggle, and a meaningful connection is impossible without empathy and cooperation (67).

Empathy is essential for lasting social change and sustainable collaborative actions to disrupt disparities. Empathetic traits are predictors of conflict-free decision-making and interpersonal cohesion that help to accept and integrate social changes (68). In this section, we will briefly introduce the concept of empathy including its biological underpinnings as it relates to the topic of the current issue.

### The fundamentals of empathy

Empathy is considered the capacity to understand, feel and assess what other individuals experience in its context (69). Empathy encompasses many motor, affective-emotional, social, cognitive, and behavioral processes which are mediated by the activity in a set of neural areas, including the temporal pole, the precuneus, the ventromedial prefrontal cortex, the bilateral angular gyrus, the amygdala, the insula, and the sensorimotor cortices (69).

### A primer on empathy in the context of social interactions

Success in social interactions is critical for promoting leadership in improving health (70). Social interactions in humans are rooted in different implicit and intrapsychic social cognitive skills (69). Social cognition studies have explored the processes that underlie social and emotional perception and their integration (71): the processes that allow humans to have empathy for others, including the capacity to mimic others’ motor behaviors (motor resonance processes) (72), sharing the emotional and painful experiences of others (affective sharing) (73, 74) and understanding others’ intentions, mental and emotional states (perspective taking) (75); as well as the processes that lead to increased drive and motivation for helping others and behaving in a cooperative manner (cooperative behavior or compassion) (76).

Empathy is so essential for human communication that the lack or loss of empathy are not variants in the human behavioral repertoire; they are symptoms. Two conditions that prominently affect the ability to empathize are psychopathy and behavioral variant frontotemporal dementia (bvFTD). The harmful actions and violation of the rights of others in psychopathy is associated with a lack of empathy (77). In bvFTD, the loss of empathy results in severe problems in close relationships (78). These display that the empathic ability is crucial for our most basic human connections.

In relation to empathy, humans can also behave in cooperative and altruistic forms in certain situations (68, 79–81). Cooperation is an organizing principle in human societies (82, 83). Evolutionary explanations of cooperation were proposed including kin selection, reciprocity and group selection; the extent of the use of these mechanisms and the ability to learn from the others are suggested to be different in humans than other organisms (83). Cooperation is dependent on empathy where individuals’ cooperative capacity are affected by whether they recognize the moral view of the others (84, 85), but empathy is not the sole decisive factor (86). These behaviors are represented in a set of brain areas, including the orbitofrontal cortices and ventral striatum. Moreover, cooperation is determined by other psychological traits including motivation, drive and positive affect all of which promote well-being (87, 88).

Empathy and cooperative behaviors (sometimes referred as compassion behaviors) (76) are crucial skills for promoting leadership in brain health. Public health leaders must understand health disparities, recognize the suffering, and share an urgency to intervene. The impact of actions on improving brain health are highly determined by the level of understanding of individuals to be served. That understanding requires empathy and cooperation (87, 88).

### The role of empathy and cooperation in brain health leadership

Measuring an individual’s ability to read the emotional state of others, to be empathetic with their affective states, to infer their mental states and cooperation are vital steps in the development of adaptive forms of leadership. Individuals with high social cognitive skills can use these skills to nurture positive relationships with others. If brain health leaders can reflect on the level of empathy in their leadership decisions, they can succeed in improving the performance of their teams.

One principal form of leadership that relies on empathy and cooperative skills is the servant leadership approach (64). Understanding the emotions, actions and decisions of the people being served is crucial in the operational process of this leadership and this information is used to deploy the team in the most effective way as well as to develop strategies that best fit the target population. This approach helps the leaders elevate non-privileged staff members’ efforts (65).

In brain health, as the suffering of the patients may involve a dehumanizing process in which the patients lose their autonomy; empathy and cooperation become essential features of the medical management. Most of the time, families and close relatives are responsible for the care of the patients, which also puts family members in a vulnerable situation. In those scenarios, empathy is crucial for fitting more humanistic interventions, focused on promoting autonomy and participation, so as the cooperation skills, the ability of behaving to cover for and favor others’ needs.

Empathy and cooperation are both critical to promote brain health changes. However, those processes do not always interact in a parsimonious manner, rather paradoxical interactions may occur. Although some aspects of empathy such as the empathic concern are positively associated with motivation and orientation to help others, different domains of empathy could attenuate prosocial behaviors (89). Notably, personal distress experienced by individuals with high empathy when seeing others’ suffering could sometimes reduce their cooperative skills (90). The emotional responses (i.e., heightened sensitivity and compassion, affective empathy), may reduce the capacity to trigger behavioral motivation to cooperate and to mobilize appropriate actions for helping others (89, 91). The mentioned effect has been previously reported in health settings in which physicians and nurses could be affected by their capacity to help when they experience a high degree of distress toward other’s needs (92–94). High personal distress could affect leaders’ capacity to cope with emotional demands and organize and implement concrete actions for promoting positive changes. Considering that empathy and cooperation are critical processes to initiate actions and promote changes, leaders working in community should be prepared to deal with emotional load.

## Empathy and cooperation align with radical collaboration

For successful leadership in global brain health, we find radical collaboration essential. Radical collaboration is a term coined by Tamm and Luyet over 20 years ago, which refers to “an animated network of actors working toward a shared frame of collective action” (95). Its importance is emphasized particularly for global problems where the efforts of single entities do not suffice to overcome the depth of the problem. Radical collaboration settings leverage individuals’ interests and intrinsic motivations while grounding collaboration in freely made commitments between peers (95). By grounding themselves in partnership, peers anchor with empathetic, cooperative, and equitable scenarios, featuring a fluid approach to leadership granted by the trust. These facets of radical collaboration paint a striking alternative to the traditional corporate model by providing more opportunities for the less-privileged actors (96). Radical collaboration has been applied on a global scale by the United Nations General Assembly during the COVID-19 pandemic when responding to global crises and reaching sustainable development goals (97).

Radical collaboration could also be beneficial to tackle brain health challenges particularly in low-income countries in which specific social disparities might play a significant role in brain health (98). Leaders in brain health should establish dialog with local leaders based on empathy and cultural humility to understand the social, medical and cultural conditions that regionally determine brain health. Leaders can then draw, coordinate, and build structured plans. It is essential to recognize the unique challenges faced by each community and to address systemic issues, to effectively promote equity in access to resources and opportunities, and responsive care (99, 100).

Radical collaboration principles have also been applied to resolve issues related to global disparities in brain health. The Atlantic Fellowship for Equity in Brain Health at the GBHI is located in UCSF Memory and Aging Center and Trinity College, Dublin, and offers a year-long fellowship program to professionals including clinicians, scientists, artists, art producers, and economists from around the world (101). The program is sponsored by the Atlantic Institute and run in collaboration with other programs that promote fairer, healthier, and inclusive societies. By embracing diverse perspectives of fellows coming from high-, middle-, and low-income countries within the processes of problem-solving including idea generation, solution finding, and innovation, and practicing empathy and cultural humility, future leaders for equity in brain health facilitate equitable and non-hierarchical interactions, which are critical to overcome misunderstanding, low trusts, interpersonal and political conflicts, facilitate active and lasting collaborations, and stimulate positive societal change.

## Conclusion

Disparities exist on different levels between and within countries, affecting brain health from birth to death. Cultural humility is essential in addressing disparities across populations, given their diverse needs and access and the impact that bias and lack of understanding can have on systems and the consequent perpetuation of inequalities. Empathy and its conscious utilization in daily interactions and collaborative models are required for cultural humility, cooperation, and collaboration. It may appear straightforward in the era of globalization but the recorded persistent and growing health and social disparities tell a different story and call for a more active approach.

## Data availability statement

The original contributions presented in the study are included in the article/supplementary material, further inquiries can be directed to the corresponding author.

## Author contributions

This manuscript was prepared as a statement paper following the authors joint presentation at the GBHI Annual Meeting at 2022. All authors contributed to the design and the content of the above mentioned presentation and the manuscript. FF, CL, TM, HS, and YA wrote the first draft. YA wrote the first draft and edited the final manuscript. YA supervised the authorships and the writing of the manuscript. All authors revised the working drafts. All authors contributed to the article and approved the submitted version.
